# Prescription drugs and mitochondrial metabolism

**DOI:** 10.1042/BSR20211813

**Published:** 2022-04-12

**Authors:** Cameron A. Schmidt

**Affiliations:** 1 East Carolina Diabetes and Obesity Institute, East Carolina University, Greenville, NC, U.S.A.; 2 Department of Physiology, Brody School of Medicine, East Carolina University, Greenville, NC, U.S.A.

**Keywords:** Metabolism, mitochondria, Pharmacology, Prescription Drugs, Xenobiotics

## Abstract

Mitochondria are central to the physiology and survival of nearly all eukaryotic cells and house diverse metabolic processes including oxidative phosphorylation, reactive oxygen species buffering, metabolite synthesis/exchange, and Ca^2+^ sequestration. Mitochondria are phenotypically heterogeneous and this variation is essential to the complexity of physiological function among cells, tissues, and organ systems. As a consequence of mitochondrial integration with so many physiological processes, small molecules that modulate mitochondrial metabolism induce complex systemic effects. In the case of many commonly prescribed drugs, these interactions may contribute to drug therapeutic mechanisms, induce adverse drug reactions, or both. The purpose of this article is to review historical and recent advances in the understanding of the effects of prescription drugs on mitochondrial metabolism. Specific ‘modes’ of xenobiotic–mitochondria interactions are discussed to provide a set of qualitative models that aid in conceptualizing how the mitochondrial energy transduction system may be affected. Findings of recent *in vitro* high-throughput screening studies are reviewed, and a few candidate drug classes are chosen for additional brief discussion (i.e. antihyperglycemics, antidepressants, antibiotics, and antihyperlipidemics). Finally, recent improvements in pharmacokinetics models that aid in quantifying systemic effects of drug–mitochondria interactions are briefly considered.

## Introduction

### The mitochondrial energy transduction system

**Figure 1:**
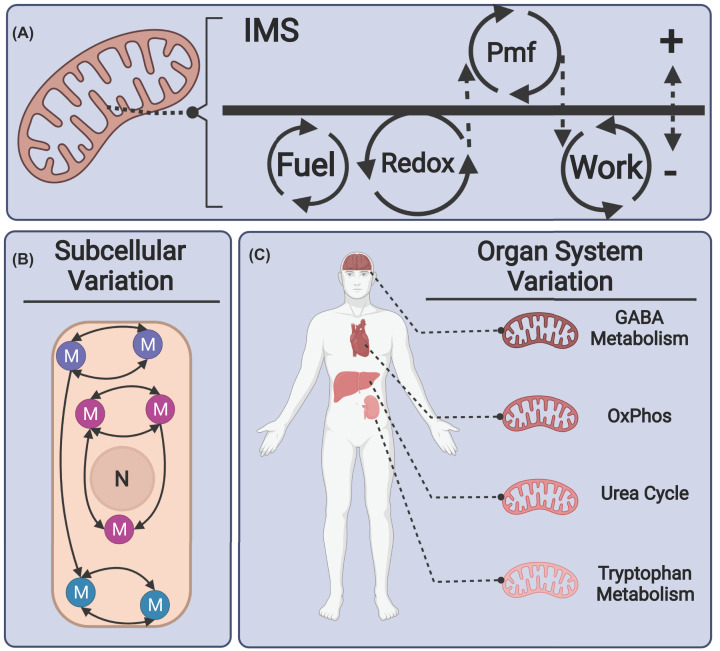
Mitochondrial structure and function are anisotropic within cells and heterogeneous among tissues (**A**) Schematic of the mitochondrial energy transduction system. Energy from fuel combustion is transduced through the redox reactions of the ETS to generate a pmf. The pmf is dissipated at steady state to drive endergonic work processes such as oxidative phosphorylation and metabolite transport against concentration and charge gradients. ‘IMS’ denotes the intermembrane space. (**B**) Mitochondria (M) vary in phenotype within cells (indicated by different colors and connections), and spatial distribution of mitochondrial functions may contribute significantly to cell physiology. Cell nucleus is labeled (N). (**C**) Mitochondrial phenotypes vary across tissues and demonstrate features (e.g. functional proteomes) that reflect the metabolic demands of the source tissue. In this example, ontological protein enrichment of tissue-specific metabolic pathways in mitochondrial isolated from brain, heart, liver, and kidney are shown. Information adapted from Johnson et al. [13].

Mitochondrial energy transduction fundamentally consists of exergonic fuel combustion coupled with various endergonic work processes (Figure 1A). Mitochondrial work output is largely dedicated to oxidative phosphorylation of adenosine diphosphate (ADP) to adenosine triphosphate (ATP), but also includes other energy coupled reactions as well [1,2]. The energy transferred from combustion is ‘stored’ in an intermediate form as an electrochemical potential across the inner mitochondrial membrane. This is denoted as proton motive force (pmf) and is derived from active proton pumping by respiratory complexes I, III, and IV of the electron transfer system (ETS). The pmf is also influenced by the passive equilibration of charged ions across the membrane, which means that the membrane potential consists of both a diffusive component (ΔpH) and an electrical component (ΔΨ) [3]. Though a more detailed discussion of quantitative bioenergetics is outside of the scope of this review, this subject has been well summarized previously [4,5]. Additionally, several analog model systems have been described that frame mitochondrial energy transduction in linear terms, allowing straightforward quantitative approximation of the behavior of the system [6–8].

### Mitochondrial structure and function are anisotropic within cells and heterogeneous among tissues

Striking experimental evidence generated over the last few decades demonstrates that the subcellular spatial distribution of distinct mitochondrial phenotypes plays an important role in the functional differentiation of cells (Figure 1B). For example, pericapillary and intramyofibrillar mitochondria in skeletal myofibers maintain distinct patterns of ETS enzyme expression that facilitate local generation and remote dissipation of membrane potential in order to overcome diffusion rate limitations during contraction [9]. In another example, perigranular, perinuclear, and subplasmalemmal mitochondria in pancreatic acinar cells exhibit differential activation by spatiotemporally distinct Ca^2+^ depolarization events, which are linked with fine-tuned control of exocytotic secretion [10]. Though the understanding of mitochondrial spatial heterogeneity is still in its infancy, it is gaining traction as methods improve for studying genetic and phenotypic variables (e.g. mtDNA heteroplasmy, mitochondrial proteome, post-translational modifications, and supercomplex stoichiometries) (for review, see Aryaman et al. [11]).

In addition to subcellular anisotropy, mitochondria isolated from different tissues exhibit functionally distinct features that reflect the physiological demands of the source tissues (Figure 1C). For example, hundreds of (largely nuclear encoded) proteins differ by expression pattern among mitochondria isolated from rat brain, kidney, liver, and heart [12]. Many of these proteins segregate ontologically by known physiological functions of the tissues (e.g. GABA metabolism in brain, urea cycle in liver, oxidative phosphorylation in heart) [13]. Additionally, mitochondria isolated from mouse heart, skeletal muscle, and liver demonstrate distinct fuel preferences and thermokinetic range that are concomitant with the predicted energetic demands of the source tissues [14]. For a more thorough discussion of distinct energetic profiles of mammalian tissues and cell types, the book chapter by Moreno-Loshuertos and Fernandez-Silva is recommended [15].

### Variation in mitochondrial metabolism has a complex relationship with whole organism phenotype

The complexity of the consequences of variation in mitochondrial metabolism are typified by mitochondrial diseases that arise from mutations in mitochondrion- or nucleus-encoded genes (recently reviewed by Thompson et al. [16]). Some mitochondrial diseases exhibit relatively uniform symptoms, but with a broad range of underlying causes. For example, Leigh syndrome involves bilateral focal lesions in the central nervous system and lactic acidosis in young children [17]. Leigh syndrome is associated with >75 distinct monogenic causes, including mutations in both mtDNA- and nDNA-encoded genes that ultimately result in ETS impairment [18]. Other mitochondrial diseases may exhibit complex phenotypic outcomes from a single underlying cause. For example, pyruvate dehydrogenase mutations (typically E1α subunit) result in a wide variety of clinical manifestations in young children, but models that predict these phenotypic outcomes are limited [19].

Together, these points introduce the immensity of the challenges associated with understanding the relationships between factors that affect mitochondrial metabolism and associated physiological outcomes. In the case of xenobiotics (i.e. natural products as well as approved or investigational prescription drugs), mitochondrial exposure to a given compound will of course be subjected to pharmacokinetics variables such as tissue distribution and metabolism. However, for cells/tissues that are exposed to xenobiotics at sufficient concentration and time to induce effects, the physiological outcomes may be unpredictable due to variation in mitochondrial phenotypic distributions over a range of biological scales (e.g. cells to tissues). The line between mitochondrial therapeutic effect and impairment is a continuum, and drug interactions may manifest as part of a therapeutic mechanism in one organ system but induce adverse reactions in another. The purpose of this article is to review historical and recent advances in the understanding of interactions between mitochondria and common approved and investigational prescription drugs. First, documented effects of non-prescription drug xenobiotics (e.g. synthetic or natural products) on components of the mitochondrial energy transduction system are discussed with the hope of providing qualitative ‘models’ that can be used to conceptualize and classify canonical drug interactions. Second, a brief review of available *in vitro* screening assays is presented, and a few specific drug classes are discussed. Finally, some limitations of current pharmacokinetics and pharmacodynamics models are examined, and potential areas of additional research focus are highlighted.

## Xenobiotics as models for potential prescription drug effects on mitochondrial metabolism

**Figure 2:**
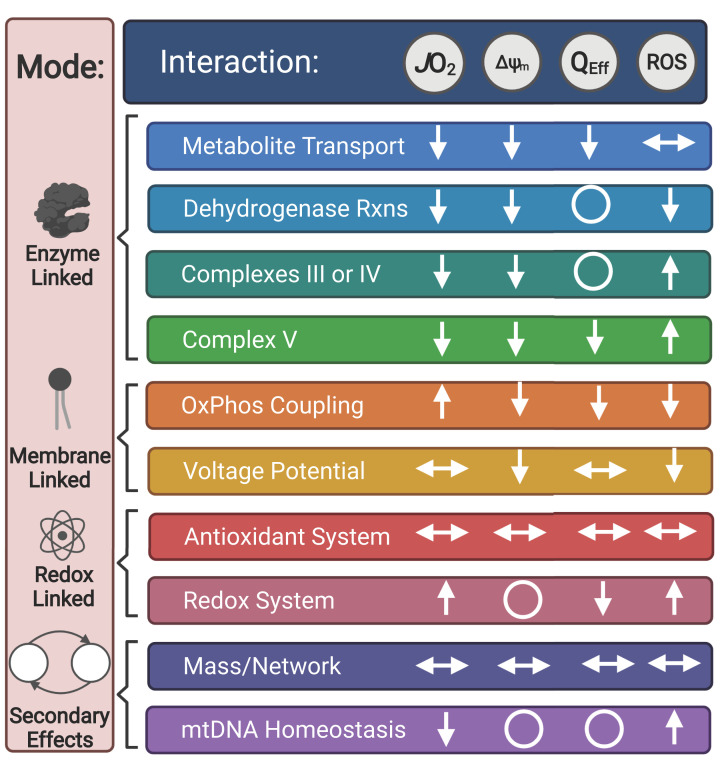
Functional ‘modes’ of xenobiotic interactions with mitochondrial metabolism and accompanying changes in empirical measurements Diagram relating specific modes of xenobiotic interactions with effects on the mitochondrial energy transduction system. Empirical measurements in isolated mitochondria and/or subcultured cells are emphasized. JO_2_ is mitochondrial respiration. ΔΨ_m_ is the mitochondrial inner membrane potential. Q_Eff_ is the apparent coupling efficiency of OxPhos, typically determined from titration of an ATP synthase inhibitor. ROS is reactive oxygen species (e.g. rate of H_2_O_2_ production). Arrows indicate anticipated direction of change upon interaction. Up/down arrows indicate increase or decrease, respectively. Horizontal double arrows indicate variable change. Circles indicate no change. Abbreviation: Rxns, Reactions.

Xenobiotic compounds such as plant- or bacteria-derived secondary metabolites are common sources of prototype drugs. Numerous interactions of xenobiotics with mitochondrial metabolism have been documented and many of these compounds are used for experimental study of mitochondrial physiology. These interactions are diverse and can be empirically measured and classified by their impact on specific elements of the mitochondrial energy transduction system through measurements of respiration rate or other parameters (e.g. membrane potential, H_2_O_2_ production, etc). Thus, the mechanistic details of xenobiotic exposure on mitochondrial metabolism may serve as useful models for the prediction or classification of prescription drug effects. Such an approach may lead to new hypotheses by comparing chemical structural similarities or other predictive parameters. In the following subsections, some specific mechanistic modes are discussed that may aid in categorizing interactions between xenobiotics and mitochondrial metabolism, particularly as they relate to empirical assays performed in isolated mitochondria and/or subcultured cells (Figure 2).

### Interactions with metabolite transport

The outer mitochondrial membrane is permeable to solutes of up to approximately 4–6.8 kDa in molecular mass, due to the presence of voltage-dependent anion channels [20]. The inner mitochondrial membrane is generally impermeable to solutes and metabolite transport is mediated by a mitochondrial solute carrier (MC) family of proteins (SLC25), of which 53 unique family members have been identified in humans [21]. Many of these carriers control metabolic reaction energetics and kinetics by altering the steady-state concentrations of reaction intermediates. Though many catalyze obligatory transport mechanisms that are neutral to the electrical and pH gradients, others dissipate these gradients as part of their energetic mechanism [2,22]. Importantly, shared sequence/structure features among MCs are necessary for transport function, suggesting that many of these transporters have common mechanisms (reviewed by Ruprecht and Kunji [21]).

MC transport generally involves an alternating gated mechanism that exposes a highly specific single substrate-binding site on one side of the inner mitochondrial membrane at a given time [21]. This mechanism of transport relies on substrate binding-dependent disruption of a conserved salt bridge network resulting in conformational change [21]. The best studied xenobiotic interactions with an MC family member are those of carboxyatractyloside and bongkrekic acid with the ADP/ATP carrier protein (AAC; a.k.a. adenine nucleotide transporter ANT) [23]. These molecules inhibit transport by fixing the conformation toward the intermembrane space or matrix, respectively.

The effects imposed by inhibition of MCs are extremely diverse due to the broad function of the MC family overall. The primary effect of inhibition of MC members is the compartmentation of metabolic intermediates [24]. Compounds that inhibit ETS-linked substrate transport will inhibit respiration in intact cells or isolated mitochondria, and this effect will not be recoverable by the addition of an uncoupler (i.e. a molecule that facilitates dissipation of pmf independent of oxidative phosphorylation of ADP; e.g. FCCP-carbonyl cyanide *p*-trifluoromethoxyphenylhydrazone) [4,25]. Alternatively, compounds that inhibit pmf-dissipating processes will also inhibit respiration but this effect will be recoverable by the addition of an uncoupler [25].

### Interactions with mitochondrial dehydrogenase reactions

The mitochondrial ETS is fueled by reducing equivalents produced by several dehydrogenase reactions that take place in both the matrix and intermembrane space. These reactions can be generally classed by their use of either NAD^+^/NADH or FAD/FADH_2_ redox pairs as cofactors and include reactions that oxidize organic ‘fuel’ molecules to provide reducing equivalents for the ETS (e.g. pyruvate dehydrogenase and isocitrate dehydrogenase) as well as reactions that reduce ubiquinone directly (e.g. NADH oxidoreductase, α-glycerophosphate dehydrogenase, and succinate dehydrogenase (SDH)). Small molecule interactions with mitochondrial dehydrogenases are as diverse as the enzymes themselves. A theme that emerges among these interactions is the competitive inhibition by metabolite analogs that block access of biomolecules to specific binding sites.

As examples, acyl phosphonates (pyruvate analogs) inhibit pyruvate dehydrogenase complex activity in plants and animals [26,27]. Inhibitors of respiratory complex I NADH:ubiquinone oxidoreductase activity are numerous and fall into three general categories: (1) quinone antagonists (e.g. piericidin), (2) semiquinone antagonists (e.g. rotenone), and (3) quinol antagonists (e.g. stigmatellin) [28]. Mitochondrial α-glycerophosphate dehydrogenase is inhibited by a class of benzimidazole-phenyl-succinimide compounds, but the specific binding domain(s) have not been identified [29]. Interestingly, reports of inhibitors that act on mammalian SDH are sparse. A class of carboxamide compounds have been used as broad-spectrum fungicides that inhibit at the quinone-binding site of SDH, and closely related carboxin (carboxaniline) has been shown to inhibit mammalian SDH activity *in vitro* [30,31]. Competitive inhibition of the succinate-binding site by malonate has also been used as an inhibitor in *in vitro* experiments involving SDH, and esterified or acylated malonate prodrugs have been used for delivery of malonate across the plasma membranes of intact cells [32].

Inhibition of specific dehydrogenase enzyme activities will affect respiration to varying degrees depending on tissue/cell type of origin and supported substrate conditions. Notably, respiratory inhibition should not be recoverable by addition of an uncoupler because respiration would be supply limited in this scenario [4]. In isolated mitochondria, respiration may be recovered by addition of substrates that circumvent the inhibited site (e.g. rescue of rotenone inhibition by addition of succinate). In intact cells, the effects on respiration may be more dramatic compared with isolated mitochondrial preparations due to mixed patterns of substrate oxidation and product inhibition by shared intermediates among different pathways. For example, the TCA cycle shares both NADH:ubiquinone oxidoreductase activity and SDH activity, and inhibition of one will inhibit the other due to accumulation of reaction intermediates. If inhibited respiration is observed in intact cells, more specific details of the implicated site(s) can be investigated using isolated mitochondria, or permeabilized cell preparations [7].

### Interactions with respiratory complex III or IV

The cytochrome *c* reductase (complex III) reaction involves the reduction of cytochrome *c* by ubiquinol with accompanying net translocation of protons to the intermembrane space. The net reaction is a two-step process involving two quinone-binding sites (Q_o_ and Q_i_). Complex III inhibitors generally target these two sites. Antimycin A is a bis-lactone secondary metabolite of *Streptomyces* bacteria that binds the Q_i_ site, preventing ubiquinone oxidation in the Q-cycle [33]. Myxothiazol and stigmatellin are also bacterial secondary metabolites that prevent quinol oxidation at the Q_o_ site with distinct but overlapping binding pockets [34,35]. Notably, both of these compounds may also inhibit the quinone-binding site of NADH oxidoreductase [28].

The cytochrome *c* oxidase (COX; complex IV) reaction is the terminal step in the ETS that couples proton translocation with oxidation of cytochrome *c* and reduction of molecular oxygen to water. Its catalytic cycle involves three conformational states that are determined by the oxidation status of its metal redox centers: (1) fully oxidized, (2) partially reduced, and (3) fully reduced [36]. Cyanide, one of the best studied inhibitors of COX, binds in the heme–copper binuclear center in the partially reduced state [37]. Xenobiotic cyanide is released from substances found in plants, such as cyanogenic glycosides, but can also be delivered directly in the form of potassium or sodium salts. Azide is another COX inhibitor that binds the Cu_B_ site in the fully oxidized state and is also common in salt form [38,39].

Complex III or IV inhibitors induce complete impairment in respiration that is not recoverable by alternative substrates or uncoupling agents. In permeabilized cells or isolated mitochondria preparations, inhibition of respiration at complex III can be recovered by the use of an artificial substrate that reduces cytochrome *c*, such as N,N,N′,N′-Tetramethyl-*p*-phenylenediamine dihydrochloride (TMPD) [4]. Additionally, inhibition of complexes III or IV may induce a high rate of reactive oxygen species (ROS) production at different sites within the ETS [40,41]. Notably, this effect can manifest as respiration in experimental preparations, i.e. O_2_ may be reduced to H_2_O, but through an H_2_O_2_ intermediate by redox buffering enzymes rather than COX activity [42]. Under these conditions, ROS production should be sensitive to inhibition of substrate oxidation reactions that act as sources of ROS, for example, inhibition of complex I by rotenone or piericidin [7,41].

### Interactions with ATP synthase

ATP synthase (respiratory complex V) is an F-type ATPase that runs in ‘reverse’, catalyzing the pmf-dependent phosphorylation of ADP to ATP [43]. In certain contexts (e.g. some cancer cell lines) the true ‘forward’ reaction predominates, catalyzing hydrolysis of ATP in support of a chemiosmotic potential [44]. Complex V consists of two general structural units, a soluble globular F_1_ unit, and an insoluble membrane-bound F_O_ unit. Notably, the F_O_ unit is named with the subscript ‘O’ for its sensitivity to inhibition by the macrolide antibiotic oligomycin A, which is by far the most common inhibitor of complex V activity used in the study of oxidative phosphorylation. Hundreds of small molecule interactions with ATP synthase have been described (reviewed by Hong and Pedersen) [45]. Inhibitors of the catalytic F_1_ unit include α-pyrone ring containing mycotoxins such as aurovertin B and citreoviridin as well as plant-derived polyphenolics such as curcumin. Common inhibitors of the F_O_ unit include polyketide antibiotics, such as the oligomycins as well as ossamycin and apopoptolidin. Different inhibitors target different subunits within the enzyme complexes which, due to variable patterns of expression, may result in a range of sensitivities to inhibition by different cell types/lines [46].

In intact cells, inhibition of complex V typically results in reduced respiration. Notably, the magnitude of effect on respiration may vary in proportion to total respiration among cell types/contexts because the degree of coupling ‘efficiency’ of OxPhos is incomplete. For example, the fractional change in respiration induced by oligomycin A in a number of representative cell types in subculture ranges from an astonishing 30–90% [47–51]. In isolated mitochondria or permeabilized cells, inhibition of complex V may be accompanied by a hyperpolarizing effect on the transmembrane voltage potential, and the rate of respiration may be recovered by titration of an uncoupling agent. Importantly, a non-linear relationship between membrane potential and respiration rate has been demonstrated in isolated mitochondria and permeabilized cells following titration of complex V inhibitors [52]. This phenomenon is due to ‘non-coupled’ pmf dissipation mediated by other processes such as pmf-dissipating enzymes (e.g. nicotinamide nucleotide transhydrogenase), passive proton ‘leak’, and production of ROS.

### Interactions with coupling of oxidative phosphorylation

OxPhos is energetically driven by the stoichiometric coupling between respiration in the ETS and ADP phosphorylation by ATP synthase (reviewed by Zorova et al. [3]). Compounds that enhance respiration rate without a concomitant increase in phosphorylation rate are known as uncouplers. Arsenate was the first uncoupler described, and functions by forming an arsenate–ADP adduct (rather than ATP) which then spontaneously decomposes back to ADP and arsenate [53]. Protonophores are a much more common class of uncouplers. These compounds consist of hydrophobic weak acids with delocalized charge that diffuse into the more alkaline mitochondrial matrix in their electroneutral (protonated) state, then dissociate, followed by the electrophoretic expulsion of the anionic conjugate base back into the net positively charged intermembrane space where they re-protonate and begin the cycle anew (reviewed by Skulachev [54]). The kinetics of protonophore activity are modulated by the chemical features of the molecules as well as the membrane composition. Lipophilicity and p*K*_a_ of the acid functional groups are of particular importance as they affect the rate of adsorption in the membrane and dissociation of the acid form [55–57]. Notably, uncoupling action of protonophores may also be mediated via activity of membrane protein intermediates, an effect identified by Starkov et al., in which mitochondrial preparations could be ‘recoupled’ by addition of hormone analogs that likely modulate the interaction between the uncoupler and the protein intermediate [58].

Uncouplers enhance the rate of respiration under any conditions in which respiration rate is limited by OxPhos kinetics. As such, this effect will be insensitive to inhibitors of ATP synthase (e.g. oligomycin A). Uncoupled respiration is also sensitive to the addition of ETS inhibitors (e.g. rotenone or antimycin A) and accompanies measurable reduction in mitochondrial membrane potential. Protonophore activity may be distinguishable from more general ionophore activity (i.e. translocation of ions other than H^+^) by assessing membrane potential components separately (i.e. ΔΨ_m_ vs. ΔpH) [59–61]. In intact cells, mitochondrial membrane potential is proportional to the magnitude of the plasma membrane potential. Notably, many protonophore activities are not membrane specific, and may also depolarize plasma membrane potentials [62].

### Interactions with the inner membrane voltage potential

A large class of xenobiotic compounds exhibit some degree of hydrophobicity and maintain a delocalized positive charge at physiological pH, rendering them capable of diffusion through phospholipid bilayers. Some of these organic cations equilibrate across the inner mitochondrial membrane following a Nernstian distribution [63].

(1) $${\Delta \Psi }_{m}=\frac{RT}{zF}ln\left(\frac{[Cation{]}_{in}}{[Cation{]}_{out}}\right)\;$$

Due to the large electrochemical potential (ΔΨ_m_), accumulation of cation in the mitochondrial matrix compartment can be quite extensive [3], i.e. at 37°C, a ΔΨ_m_ of −180 mV may drive an approximately 1000-fold greater concentration inside the matrix relative to the cytoplasm [63]. Common vital dyes used to study mitochondria, such as tetramethylrhodamine methyl ester and Safranine O, are organic cations which allow them to be used for potentiometric quantification of the magnitude of the steady-state membrane potential [60,61]. Additionally, triphenylphosphonium cations are increasingly used to ‘target’ conjugated molecules, such as antioxidants, to the mitochondrial matrix (a.k.a. mitochondriotropics) [63,64].

Organic cations exhibit biphasic uncoupling activity and broad-spectrum respiratory inhibition, each dependent upon concentration and chemical features [65]. Uncoupling effects may be due to genuine protonophore activity when the compound contains a dissociable proton (e.g. rhodamine 19 derivatives), or may be due to induced permeability of the inner mitochondrial membrane to ions and polar uncharged solutes by molecules that lack dissociable protons such as quaternary amines (e.g. cetyltrimethylammonium) [66,67]. The inhibitory actions of organic cations may be due to competitive or non-competitive interactions with specific enzymes, which are potentiated by the high concentrations of compound that accumulate in the matrix [28,68]. Additionally, inhibitory actions of organic cations become less specific at higher concentrations or with increasing lipophilicity (e.g. longer acyl chain lengths), most likely due to detergent-like interactions with various enzymes, particularly those of the ETS [65,69]. Interestingly, SDH activity has been shown to be less sensitive to these effects compared with other mitochondrial enzyme activities, however, the properties that underlie this apparent resistance are not clear [65].

### Interactions with the antioxidant system

The two-electron transfer reaction scheme from NADH to O_2_ involves more than a dozen individual reactions and maintains a steady-state redox potential as large as 1.1 Volts [5]. Maintenance of the steady-state redox potential depends on electrons being transferred through the appropriate subsequent reaction in the network, and electron ‘leak’ may be imposed by various circumstances that favor the production of ROS such as superoxide anion radical, hydrogen peroxide, hydroxyl radical, hypochlorous acid, and singlet oxygen. ROS are further reduced in either controlled reactions with redox buffering systems (e.g. the glutathione/glutathione–disulfide system) or uncontrolled reactions with various macromolecules such as lipids, proteins, and DNA [70,71]. High rates of electron leak can be achieved in isolated mitochondria/permeabilized cell preparations, but rates are generally very low under normal physiological steady states [41].

Cells contain numerous antioxidant species as primary metabolites (e.g. lipid-soluble α-tocopherol and β-carotene, or water-soluble ascorbic acid). There are also several xenobiotic antioxidants that are known to interact with mitochondrial metabolism including a range of plant-derived secondary metabolites (e.g. resveratrol) and synthetic compounds (e.g. common food-additive butylated hydroxyanisole). Most antioxidants are phenolic compounds that work by inhibiting peroxidative processes through a few distinct mechanisms depending on their chemical features and microenvironmental conditions. These include: (1) donating a hydrogen to a peroxyl radical, (2) transferring electrons in accordance with the redox potentials of the involved species, or (3) radical scavenging [72,73]. In all cases, the defining feature of the antioxidant mechanism is that the reaction kinetics outpace the competing organic substrate oxidation reaction. Most antioxidant mechanisms involve the formation of a prooxidant, and antioxidant-regenerating reaction systems are necessary to prevent accumulation of the prooxidant as well as facilitate renewed antioxidant activity [74,75]. A major consequence of this requirement is that effects of exogenous antioxidants on mitochondrial metabolism are dependent on concentration and chemical features, and may involve seemingly paradoxical effects across similarly structured compounds including: (1) inhibition *or* stimulation of ROS production, (2) enhanced coupling *or* uncoupling of respiration, and/or (3) inhibition *or* stimulation of ETS flux [76,77]. Though there are numerous studies describing the associated effects of exogenously administered antioxidants, mechanistic studies describing the underlying chemical mechanisms are limited and are a promising area of focus for further investigation.

### Interactions with the redox system (via redox cycling)

Several species of quinone-containing small molecules participate in enzyme catalyzed one or two electron reduction, followed by subsequent re-oxidation by molecular oxygen forming ROS, which are then reduced through redox buffering reactions. The net reaction comprises a redox cycle that provides an energetically favorable path for continuous electron leak from the mitochondrial redox reaction network under physiological conditions [78]. Some representative examples of these compounds are the broad-spectrum herbicide paraquat and the vitamin K metabolite menadione, both of which are often used in the experimental study of mitochondrial ROS production [79–81]. Redox cycling reactions are dependent upon diaphorase enzyme activities, such as those that oxidize NAD(P)H. There are four measurements that, when made together, can confirm the activity of a redox cycling agent: (1) the rate of oxidation of NADPH by redox buffering systems, (2) measurement of oxygen consumption, (3) detection of the semiquinone intermediate, and (4) rate of ROS production (e.g. H_2_O_2_) [78]. Stimulation of respiration by redox cycling agents may also exhibit insensitivity to certain inhibitors (e.g. rotenone or cyanide), but this effect will vary among different agents depending on diaphorases involved in catalyzing the cycle [82].

### Agents that influence mitochondrial mass or network architecture

Positive correlation between respiratory kinetics and mitochondrial mass has been demonstrated both *in vivo* and *in vitro* [83,84]. Steady-state cellular mitochondrial mass is governed by a dynamic life cycle that balances mitochondrial biogenesis and lysosomal degradation through mitophagy in close association with fission and fusion of reticulated mitochondrial networks [85–87]. Because these processes are organized through regulatory enzyme systems, small molecules that interfere with these systems could (presumably) modulate mitochondrial function independent of direct interaction with mitochondrial metabolism. Screening studies have identified novel chemical agents that influence activities of biogenic regulatory proteins such as PGC-1α (e.g. ZLN-005 and SR18292) and AMPK (e.g. 5-hydroxystaurosporine) [88–90]. Novel inhibitors of mitochondrial fission/degradation in mitophagy have also been identified that are thought to target GTPase activity such as dynamin and DRP-1 (e.g. Dynasore and mdivi-1) [91,92].

Notably, screening protocols that have been leveraged to identify these compounds were not designed to distinguish between direct and indirect effects on mitochondrial metabolism [88–92]. Indirect interactions through biogenesis or degradation have been shown to require a period of several hours *in vitro*, and thus, should reflect timescales that differ from direct interactions with mitochondrial energy transduction which are typically much faster [93]. Supporting evidence for the notion that any of the aforementioned compounds function purely through indirect effects is limited, and follow-up studies have demonstrated some direct effects including inhibitory activity against ETS enzymes and antioxidant activities [94,95]. This highlights that caution should be taken when classifying xenobiotics as indirect effectors of mitochondrial metabolism unless the possibility of direct effects has been ruled out.

### Inhibitors of mtDNA replication or translation

Mitochondria maintain distinct genomes and require energy-dependent protein import from the cytosol in support of complex cycles of fusion, fission, replication, and degradation [87,96]. The vast majority of the mitochondrial proteome is nuclear encoded, and functional discordance between the two genomes induces complex adaptive responses [97–99]. Impairment of mtDNA homeostasis can cause catastrophic phenotypic manifestations, which follow from a variety of underlying mitochondrial functional limitations [97,100,101]. Cultured cells can be experimentally depleted of mtDNA through selective use of chelating agents such as ethidium bromide (denoted as ρ^o^ cells) [102]. The specific functional implications of mtDNA depletion may vary, but are generally characterized by reduced ‘basal’ and ‘maximal’ respiration rates as well as increased steady-state ROS production [103]. An interesting primary effect of mtDNA loss, initially discovered in the 1980s, is the development of specific auxotrophies (e.g. obligate requirements for exogenous uridine or pyruvate) [102]. Further examination of these effects in other cell types have established the importance of mitochondrial energy transduction in metabolite biosynthesis, highlighting the importance of the non-oxidative phosphorylation-related functions of mitochondria [104–106].

## Prescription drugs and mitochondrial metabolism

### Insights from reported drug screens

High-throughput screening assays for drug effects on mitochondrial metabolism are primarily performed for one of the two purposes: (1) identifying idiosyncratic organ-specific toxicities for novel or existing drugs—a practice which became prevalent following high-profile market withdrawal of several drugs in the late 1990s (e.g. troglitazone and cerivastatin) [107] and (2) early hit discovery for compounds intended to enhance mitochondrial function or improve functional impairments that accompany diseases such as neurodegeneration or cancer (reviewed by Andreux et al. [108]). The distinction between the two types of screens is largely attributable to interpretation, as the outcome measures are often the same (e.g. cellular ATP concentrations measured by chemiluminescence) [109–111].

Screening assays are generally performed using subcultured cell lines derived from various species/tissues and include either single or multiparametric assessment of mitochondrial functional/morphological parameters such as rate of respiration, cellular ATP concentrations, diaphorase activity, inner mitochondrial membrane potential, or mitochondrial mass/network architecture [109–113]. To enhance sensitivity of the assays, cells are sometimes incubated with ‘non-glycolyzable’ carbohydrate sources (a.k.a. nutrient sensitization) to force a reliance on mitochondrial oxidative phosphorylation [114–116]. In addition to direct assessment in subcultured cells, other studies have taken an *in-silico* approach, combining machine learning modalities with quantitative structure–activity relationship analysis (QSAR) [117,118]. These methods have been used for large-scale classification of molecular properties associated with mitochondrial toxicities, of which the most predictive descriptor was notably determined to be a calculated partition coefficient (SLogP) between 4 and 9 (>25% and <1% negative values) [117]. This is further supported by the observation that approximately 50% of drugs identified by the other *in vitro* screening studies described above exhibit calculated partition coefficients between 4 and 9, and only 4% were negative values (Supplementary Table S1). This association indicates that membrane permeability is a key predictor of direct effects on mitochondrial metabolism.

**Figure 3:**
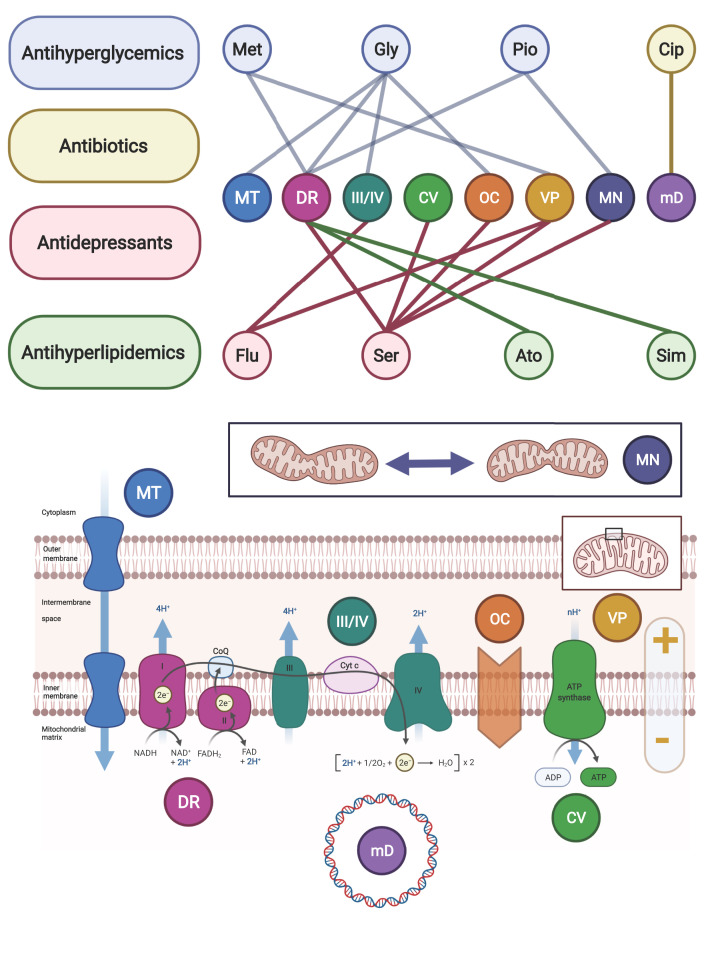
Representative prescription drug interactions with mitochondrial metabolism Bipartite graph with edges highlighting documented interaction ‘modes’ of some representative prescription drugs with mitochondrial metabolism. Abbreviations: Ato, Atorvastatin; Cip, Ciprofloxacin; Gly, Glyburide; Flu, Fluoxetine; Met, Metformin; Pio, Pioglitazone; Ser, Sertraline; Sim, Simvastatin. Interaction ‘modes’ are related to those highlighted in Figure 2 and mapped in this figure on to a diagram of the mitochondrial energy transduction system. Abbreviations: CV, complex V (ATP synthase); DR, dehydrogenase reaction(s); mD, mtDNA homeostasis; MN, mitochondrial mass/network; MT, metabolite transport; OC, oxidative phosphorylation coupling; VP, voltage potential; III/IV, complex III/IV.

To ‘narrow down’ specific drug categories for additional brief discussion, a large multiparametric *in vitro* screening dataset was mined against drugs listed in a recently published list of over 200 approved or investigational prescription drugs [111,119]. The chosen screening study, reported by Wagner et al., was ideal for this purpose because the data are publicly available, and the normalized z-scores provided bidirectional changes which allowed the data to be interpreted as either ‘enhancing’ or ‘impairing’ mitochondrial metabolism [111]. Several representative groups were identified for further discussion by the observed effects on the cells in the screening study, by first-line therapy indications and adverse reactions, and by documented effects on mitochondrial metabolism determined from additional literature review. The selected classes include: antihyperglycemics, antidepressants, antibiotics, and antihyperlipidemics (Figure 3).

### Antihyperglycemics

Biguanide drugs are guanidine compounds that are widely used in the treatment of type II diabetes mellitus. Metformin (DrugBank accession: DB00331), the most commonly prescribed biguanide, has been implicated in several physiological processes including stimulation of peripheral glucose uptake, inhibition of hepatic gluconeogenesis, and reduction in insulin production (reviewed by Yerevanian and Soukas [120]) [121]. Metformin exerts biphasic effects on mitochondrial metabolism. For example in rat permeabilized muscle fibers *in vitro*, metformin inhibits H_2_O_2_ production in the μM range, but inhibits respiration in the mM range [122]. Metformin is also a non-competitive inhibitor of respiratory complex I in the mM concentration range (i.e. does not interfere with either NADH or ubiquinone binding), as evidenced in isolated human and bovine heart mitochondria as well as primary human hepatocytes [123–125]. Notably, metformin and other biguanides are predicted cations at physiological pH, and their IC_50_ values for complex I activity decrease linearly with lipophilic tendency [123]. In mice, metformin accumulates in the kidneys, adrenal glands, pancreas, and liver [126]. Interestingly, significant accumulation in the digestive tract has also been noted in both mice and humans following administration both orally and parenterally [126,127]. The relative contribution of this effect on Metformin’s antidiabetic activity is not clear, though there is evidence that exposure of the digestive system to high concentrations of metformin may contribute to its glucose regulating effects (at least in part) through an intestinal–hepatic futile cycle [128,129].

Sulfonylurea drugs are also prescribed for management of type II diabetes mellitus. Glyburide (a.k.a. Glibenclamide; DrugBank accession: DB01016) is commonly prescribed in conjunction with metformin and inhibits ATP-sensitive potassium channels on pancreatic β-cells resulting in insulin secretion. Patch clamping of human dermal fibroblast mitochondrial inner membranes indicated that glyburide (30 μM) diminished K_ATP_ channel opening [130]. Glyburide also inhibited pyruvate carboxylase supported flux in rat liver isolated mitochondria (IC_50_ = 63.3 μM), while increasing pyruvate supported respiration in the absence of ADP, indicating an uncoupling activity [131]. In rat H9C2 cells (a cardiomyoblast cell line), glyburide impaired mitochondrial respiration in live-intact cells and complex I–III activity in protein homogenates in the μM concentration range [132]. However, there does not appear to be any direct evidence that glyburide alters mitochondrial metabolism in intact β-cells, and a mouse β-cell line depleted of mitochondrial DNA remained sensitive to glyburide-stimulated insulin secretion [133]. However, mitochondrial effects in other tissues have been of interest due to apparent cardio and renal toxicity of this drug, as well as compounding evidence regarding the function of mitochondrial K_ATP_ channel activities (reviewed by Pereira and Kowaltowski [134]). In humans, glyburide accumulates in highly perfused organs such as liver and kidney [135].

Thiazolidinedione drugs are used for the treatment of type II diabetes mellitus and increase insulin sensitivity through a putative mechanism involving activation of PPARs which may influence mitochondrial mass or network architecture (eeviewed by Nanjan et al. [136]). Pioglitazone (DrugBank accession: DB01132), a common thiazolidinedione, is like other antidiabetic drugs discussed so far in that its therapeutic and adverse effects are likely mediated through its impact on mitochondrial metabolism. Pioglitazone stimulates transactivation of purified human PPARs (1–10 μM range) [137]. However, as mentioned in the previous section, specific effects of xenobiotic compounds that alter transcriptional regulation of mitochondrial metabolism can be difficult to separate from direct effects on the energy transduction system. In mouse liver mitochondria, pioglitazone binds and inhibits complex I activity (1–10 μM), and when administered *in vivo* or in HEPG2 cells, stimulates transcription of nucleus-encoded mitochondrial genes (10 mg/kg/day and 10 μM, respectively) [138]. In humans swith type II diabetes mellitus, 12-week treatment with pioglitazone increased insulin sensitivity and glucose utilization, but did not alter muscle mitochondrial function [139]. However, pioglitazone did alter transcription of nucleus-encoded mitochondrial genes in human adipose tissue suggesting the effects may be mediated by tissues other than muscle [140].

### Antibiotics

Antibiotics are a large category of drugs that are generally classed as either bacteriostatics or bactericidals. Because mitochondria share putative homologous origins with prokaryotes, some of the mechanisms that underlie antibiotic activities likely also influence mitochondrial metabolism. Several classes of antibiotics including quinolones, β-lactams, and aminoglycosides inhibit respiration, induce a pro-fission state, and stimulate ROS production in mammalian cells (10–25 μg/ml) and in mice (12–28 mg/kg/day) [141]. Treatment with the antioxidant N-acetylcysteine or triphenylphosphonium conjugated ubiquinone (MitoQ) reduced some of these effects.

Fluoroquinolone bactericidal drugs are well-studied in the context of their interactions with mitochondrial metabolism, and ciprofloxacin (DrugBank accession: DB00537) is one of the most widely prescribed antibiotics worldwide. Ciprofloxacin inhibits topoisomerase isoforms and DNA gyrase resulting in disrupted ligase activity, which induces double-stranded breaks and death. Ciprofloxacin inhibited mtDNA replication in mouse C2C12 myoblasts in a topoisomerase II-dependent manner and interfered with both proliferation and differentiation to myotubes (40 μg/ml) [142]. In human primary T cells, prolonged treatment with ciprofloxacin reduces complex I activity though repression of catalytic subunit expression, resulting in suppression of inducible IL-2/4 expression [143]. There does not appear to be any direct evidence that ciprofloxacin directly interferes with mitochondrial respiratory system or other non-mtDNA replication related processes directly. However, alteration of mtDNA homeostasis can result in complex physiological effects. For example, similar effects are typified by the mtDNA polymerase γ (PolG) mutator mouse model [101]. In those mice, complex phenotypic patterns such as progeria and cardiac insufficiency can be traced to downstream adaptive responses to complex I deficiency that results from mtDNA loss-of-function mutations [103]. However, the extent to which antibiotic treatment can induce such impairments in humans requires more investigation. For additional information regarding antibiotic interactions with mitochondrial metabolism, a recent review by Suarez-Rivero et al. is recommended [144].

### Antidepressants

Selective serotonin reuptake inhibitors (SSRIs) are a class of drugs prescribed for major depressive disorder and other anxiety disorders. SSRIs are benzenoid compounds such as tametralines (sertraline; DrugBank accession: DB01104) and trifluoromethylbenzenes (fluoxetine; DB00472). Notably, both examples are predicted organic cations at physiological pH, suggesting that they may distribute inside the mitochondrial matrix in coordination with the voltage gradient if the membrane is permeable to them. The role of mitochondria in major depressive disorder, and the influence of SSRIs on mitochondrial function, remain under debate (recently reviewed by Allen et al. [145]). These relationships are like those of biguanide drugs and mitochondria in diabetes, in that the disease may or may not be associated with underlying deficiencies in mitochondrial metabolism, and drug therapies may improve mitochondrial impairments but may also induce adverse effects through their interactions with mitochondria in other organ systems or at high concentrations that result from individual pharmacokinetic variation.

In a study involving a β-amyloid induced paralysis model in *Caenorhabditis* *elegans*, sertraline treatment lengthened the time to paralysis via mild uncoupling activity, ATP depletion, and stimulation of PINK-1-dependent mitophagy (5–25 μM) [146]. This highlights that the benefit of mitochondrial interactions with some prescription drugs may be derived from the mild toxicity that the drugs induce. A concept that echoes some of the debate regarding mechanisms underlying metformin action in diabetes discussed earlier. In rats treated with 3-nitropropionic acid, to induce Huntington’s disease-like symptoms, treatment with sertraline (10 mg/kg/day) attenuated motor dysfunction and preserved brain mitochondrial enzyme activities, an effect attributed to antioxidant function [147]. As also noted in other drug–mitochondria interactions, some of sertraline’s benefits in the nervous system may occur concomitantly with adverse reactions in other organs. For example, sertraline is associated with hepatotoxicity, and in isolated primary rat hepatocytes, sertraline (25–37.5 μM) inhibited complexes I and V activity, uncoupled respiration, stimulated mitochondrial permeability transition, and induced cellular damage [148].

Fluoxetine is another common SSRI that is also being investigated as an antineoplastic agent. Fluoxetine inhibits OxPhos, increases cytosolic calcium, and stimulates necrosis in various cancer cell lines (∼25–100 μM) [149]. In the rat hippocampus, fluoxetine treatment (15 mg/kg/day) stimulates adaptive mitochondrial proteomic alterations that reflect energy stress [150]. In the mouse frontal cortex, fluoxetine treatment (15 mg/kg/day) negatively impacts respiratory complexes III and IV activities, and induces adaptive metabolomic profile changes that are canonically associated with energy limitation [151]. Interestingly, nutritional status (e.g. overfeeding) has well-demonstrated adaptive effects on mitochondrial function, but very little is known regarding how these kinds of environmental variables may influence drug interactions with mitochondrial metabolism. In some cases, this effect may even unmask unforeseen therapeutic potential, for example, fluoxetine reverses several parameters related to altered mitochondrial functional impairments in the rat hypothalamus that follow from neonatal overfeeding [152].

### Antihyperlipidemics

HMG-CoA reductase inhibitors (a.k.a. statins) are a commonly prescribed class of antihyperlipidemic drug that have documented liabilities toward mitochondrial metabolism. Two commonly prescribed statins are simvastatin (DrugBank accession: DB00641) and atorvastatin (DrugBank accession: DB01076). Simvastatin is a δ-valerolactone compound derived from the fermentation products of *Aspergillus* *terreus*, and atorvastatin is a fully synthetic diphenylpyrrole compound. In mitochondria isolated from a human umbilical vein endothelial cell line, atorvastatin inhibited both succinate/rotenone and malate supported respiration in a calcium-dependent manner (50–100 μM) [153]. In isolated human platelets and a human hepatocellular carcinoma line, respiratory inhibition by atorvastatin was improved by exposure to a succinate prodrug, suggesting that in intact cells the inhibitor effects on complex I may predominate [154]. Both atorvastatin and simvastatin induced de-differentiation and increased ADP/ATP concentration ratios in primary human myofibroblasts [155]. In rats, treatment with atorvastatin for 20 days (10 mg/kg/day) decreased ATP concentrations and induced adaptive reduction in mitochondrial complex I activity in cardiac and renal tissues [156].

Statin compounds are lipophilic and are either uncharged or negatively charged under physiological conditions, thus, the distribution of statins within mitochondria of most tissues is generally assumed not to be substantial [157]. However, first pass organs and those that are metabolically active (e.g. heart) may be sensitive to exposure to the active hydroxy acid forms of statins. Notably, statin use is associated with new-onset diabetes as well as deteriorated glycemic control in patients with existing diabetes [158,159]. This effect is likely mediated through mitochondrial liabilities, as atorvastatin inhibits respiratory chain enzyme activities and induces swelling in mitochondria isolated from rat pancreas [160]. Additionally, atorvastatin treatment (10–100 ng/ml) reduced glucose stimulated insulin secretion accompanied in isolated human pancreatic islets and cultured rat INS-1 cells; an effect that was accompanied by reduced ATP production rates and reduced respiratory complexes I, II, III, IV, and V protein concentrations [161]. Interestingly, pravastatin (DrugBank accession: DB00175), another statin, does not similarly impact insulin secretion or respiratory complex expression [161]. Pravastatin also does not alter mitochondrial respiratory function in isolated endothelial cell mitochondria [153]. Thus, the chemical distinction between pravastatin and other statins could be important in elucidating the interactions that underly mitochondrial liabilities and should be further investigated.

### Pharmacokinetics/pharmacodynamics and mitochondria

The mechanistic underpinnings of drug interactions with mitochondrial metabolism have been largely investigated in isolated organelles, subcultured cells, and rodent *in vivo* models. These studies are useful for identifying putative associations between observed effects and chemical features but are not capable of predicting or explaining the complex effects observed in humans. This is influenced by two key factors: first, the relationships among drug doses prescribed to humans and concentrations/doses used in *in vitro* studies are not straightforward because they rarely share the same units (i.e. human dose = mass of drug/body mass/(time); *in vitro* dose = mass of drug/media volume). Second, in animal studies, the dose units are often the same, but pharmacokinetics/toxicokinetics may differ substantially by species. For example, metformin toxicokinetics in rats differ compared with humans when normalized to mass [162,163].

Clinically applied pharmacokinetics generally rely on plasma measurements and single compartment mathematical models for estimation of drug ADME (absorption, distribution, metabolism, excretion). A limitation to understanding the clinical impact of prescription drug effects on whole-body mitochondrial metabolism is that single compartment models lack resolution at the organ system and subcellular compartmental levels. Predicting the complex effects of drug interactions with mitochondrial metabolism, in both the preclinical (research) and clinical settings, will require information about the patterns of subcellular accumulation/metabolism in specific tissues as well as the mechanistic details of the drug-mitochondria interactions. Research in this area could benefit substantially from implementation of pharmacokinetic models that account for, and predict, subcellular distributions of molecules and the accompanying impacts of phenotypic differentiation among distinct tissues over time.

Recent advancements in computational methods combined with enhanced accessibility of large-scale gene expression and metabolite datasets has opened the door to broad implementation of multicompartment pharmacokinetics approaches that may be capable of overcoming some of the limitations discussed above. Physiology-based pharmacokinetics (PBPK) models have been used to predict drug interactions in industry for decades. These models quantitatively describe organ-specific intracellular drug reaction rates [164]. Additionally, genome-scale metabolic network (GSMN) fluxes can be modeled using parameter-free constrained linear optimization (e.g. flux balance analysis) [165,166]. The combined approaches (PBPK-GSMN) have been integrated to quantitatively assess multiscale blood glucose regulation in type I diabetes, as well as cellular responses during drug-induced metabolic perturbations [167,168]. A very interesting recent study used a combined PBPK-GSMN approach to examine whole-body metabolic system perturbations caused by isoniazid (DrugBank accession: DB00951), a bactericidal agent that has been implicated in drug-induced idiosyncratic liver injury with mitochondrial metabolic implications [169,170]. This study was able to quantify patterns of cell type-specific metabolite utilization rates in response to drug treatment in both fast and slow metabolizers of the drug. PBPK-GSMN approaches appear to hold much promise for quantifying and predicting the complex phenotypic relationships between prescribed drugs and mitochondrial metabolism, particularly if combined with mechanistic details of drug–mitochondria interactions identified through *in vitro* studies.

## Summary and conclusion

Mitochondrial metabolism is essential to human physiology. Because of the mitochondrial phenotypic diversity across multiple scales (e.g. from cells to tissues), interactions between drugs and mitochondrial metabolism can result in extremely complex physiological consequences. These may manifest as part of drug therapeutic mechanisms of action, or adverse reactions (or both) depending on many complex pharmacokinetics and pharmacodynamics factors. There are numerous mechanisms through which drugs can affect mitochondrial metabolism within cells. These interactions may be grouped into functional ‘modes’ based on existing models of the mitochondrial energy transduction system, which may aid in comparing or classifying specific drugs/activities. In recent decades, these models have been extended to *in vitro* screening assays and experiments in isolated mitochondria and cultured cells, which has provided a wealth of information regarding drug effects and potential adverse reactions. However, looking to the future, the most salient remaining challenge is to incorporate the detailed *in vitro* mechanisms of drug effects with the complex phenotypic outcomes that occur *in vivo*. To this end, PBPK models combined with GSMN models appear to be a promising direction, particularly for use in preclinical research where more invasive metabolic data can be obtained. Finally, the full spectrum of mitochondrial phenotypic variation over both spatial and temporal scales is very poorly understood. Both drug design and treatment practices could benefit from additional research into the principle components and regulatory systems that underly mitochondrial phenotypic variation.

## Supplementary Material

Supplementary Table S1

## Abbreviations

ADP: adenosine diphosphate
ATP: adenosine triphosphate
COX: cytochrome *c* oxidase
DRP-1: dynamin related protein 1
ETS: electron transfer system
GABA: gamma-aminobutyric acid
GSMN: genome-scale metabolic network
MC: mitochondrial solute carrier
nDNA: nuclear encoded DNA
OxPhos: oxidative phosphorylation
PBPK: physiology-based pharmacokinetics
PGC-1a: PPAR gamma coactivator 1a
pmf: proton motive force
PPAR: peroxisome proliferator-activated receptor
ROS: reactive oxygen species
SDH: succinate dehydrogenase
SSRI: selective serotonin reuptake inhibitor
